# Effectiveness of artificial intelligence mobile app-guided prevention and treatment protocols on cancer patients and their impact on healthcare workers’ competence

**DOI:** 10.1186/s12911-026-03432-1

**Published:** 2026-03-31

**Authors:** Eman A. Shokr

**Affiliations:** 1 Community Health Nursing, Faculty of Nursing, Menoufia University, Shibīn al Kawm, Egypt; 2 Faculty of Nursing, Al-Zaytoonah University of Jordan, Airport Street, Amman, Jordan

**Keywords:** Artificial intelligence, Mobile health, Oncology care, Cancer patients, Healthcare workers’ competence

## Abstract

**Background:**

Artificial intelligence-based mobile health applications are increasingly used in oncology care, mainly to support symptom monitoring and clinical workflows. However, there is limited evidence on applications that simultaneously support cancer patients through structured prevention and treatment guidance while enhancing healthcare workers’ competencies. This study aimed to assess the outcomes of an AI-based mobile application as a supportive tool for cancer patients and examine its impact on healthcare workers’ competence in routine oncology care.

**Methods:**

A quasi-experimental pre-post design was conducted with 60 cancer patients and 60 healthcare workers. The AI-based mobile application provided content on education, symptom management, and protocol-based support. Data was collected through structured online questionnaires before and after a 12-week intervention using five tools to assess usability, perceived benefits, efficiency of healthcare workers, health awareness, and selected chemotherapy-related symptoms. The same instruments were used for pre- and post-measurements without a control group.

**Results:**

Post-intervention findings indicated improved patient-reported usability of the mobile application, with most participants reporting clarity and perceived usefulness, although some reported occasional confusion or irrelevant responses. Healthcare workers demonstrated statistically significant improvements in perceived efficiency and benefits related to application use (*p* < 0.01). An increase in reported health awareness among nurses was observed. Patients also reported reductions in the severity of chemotherapy-related symptoms.

**Conclusion:**

The findings suggest that the AI-based mobile application may serve as a supportive digital tool in oncology settings by assisting patients in following prevention and treatment guidance and supporting healthcare workers’ perceived efficiency and awareness.

**Clinical trial number:**

Not applicable.

## Introduction

The growing use of technological advancements affects the healthcare sector, specifically with the application of artificial intelligence (AI). AI helps improve the quality of healthcare, enhance the efficiency of healthcare personnel, reduce the likelihood of errors, and ensure informed decision-making in healthcare [1, 2]. AI also enhances patient education, treatment processes, and trust between patients and healthcare workers.

Cancer is one of the leading causes of death worldwide, accounting for approximately 16% of all global deaths, according to the World Health Organization (WHO) [3, 4]. Despite significant advances in cancer treatment, challenges remain in cancer prevention, patient education, and the effective management of chemotherapy-related side effects. In recent years, mobile health applications utilizing artificial intelligence (AI) technologies have emerged as promising tools to support cancer care and improve patient outcomes [5].

Applying AI in cancer treatment benefits not only cancer patients, but also has the potential to improve the abilities of medical personnel. AI-based mobile application have the capacity to enhance efficiency in medical practice by providing assistance in accessing treatment guidelines and performing many other tasks. The apps give medical personnel the time to work with their patients, and patients also benefit from personal assistance, faster communication, and improved treatment adherence [6, 7].

Previous research has explored the use of AI in healthcare and cancer care. Tursynbek et al. (2024) [8] found that AI is primarily perceived as an assisting tool under human supervision. Pan et al. (2022) [9] showed that AI-based mobile application have been observed to help in patient-reported outcomes in relation to fatigue, pain, and quality of life. Samadbeik et al. [7] found that AI-assisted mobile applications help in better pain control, improved psychiatric and psychological issues, and improved wellbeing in cancer patients.

Most existing studies on AI in healthcare focus on tools for diagnosis, prediction, or symptom monitoring. Limited studies have focused on AI-based mobile applications that not only guide oncology patients through structured prevention and treatment protocols but also help healthcare workers improve their skills and knowledge. This study introduces a new AI-based mobile application for cancer care that addresses both sides: it supports patients in following their care plans and helps healthcare professionals deliver better, more competent care. By looking at how the app affects both patient outcomes and healthcare workers’ performance, this research fills a gap in the current literature and provides practical insights into how AI can improve everyday oncology practice. The current study aims to address this gap by investigating the effects of an AI-based mobile application on cancer patients’ adherence to prevention and treatment protocols and its impact on healthcare workers’ competence within routine oncology care.

### Significance of the study

Healthcare deployment of artificial intelligence will have the power to change the face of service delivery through the efficiency of health personnel, extended reach, and better patient outcomes. Cancer is a major financial and health resource burden, with costs pegged at a cumulative 500 billion USD a year, besides resource constraints and issues related to equity and quality access. AI-based mobile application have the capacity to offer customized prevention and treatment strategies for patients [5].

In addition, the lack of nurses on a global level, which was estimated to be 5.9 million in 2018 but predicted to increase to 10 million by 2030 (World Health Organization, 2020) [3], further supports the need for applications that serve to increase efficiency as well as to minimize stress as a factor in professionalism. This study, therefore, assumes great importance in proving that applications involving AI-based mobile application not only have the ability to increase patient care but also contribute to increasing healthcare professionals.

### Purpose of the study

The purpose of this study is to investigate the impact of artificial intelligence mobile app-guided prevention and treatment protocols on cancer patients and their impact on healthcare workers’ competence.

### Research hypotheses

#### H1

The utilization of artificial intelligence mobile app-guided prevention and treatment protocols has a positive impact on the clinical outcomes and overall care experience of cancer patients.

#### H2

The application of mobile app-guided prevention and treatment protocols in healthcare significantly improves the competence, efficiency, and decision-making abilities of healthcare workers.

## Methods

### Study design

A quasi-experimental design (pretest and posttest) was used to achieve the aim of the study.

### Setting

The research was conducted in a single oncology institution in Shebin Elkom, located in the capital of Menoufia Governorate, Egypt. The oncology institution provides care to more than 4000 patients each month. This institution is affiliated with the Ministry of Higher Education and represents the first point of contact for patients within the healthcare system when they have a health concern or require cancer-related investigation and treatment. This institution provides primary prevention services, including cancer prevention programs, diagnostic investigations, and therapeutic treatments such as chemotherapy and radiotherapy, in addition to outpatient services.

### Sample

The study’s target population included Group (1): A purposive sampling technique was utilized to include all accessible healthcare workers (*n* = 60) who met the following inclusion criteria: aged between 20 and 50 years, use of a smartphone, prior exposure to artificial intelligence (AI), experience with digital training, proficiency in the English language, awareness of the study’s objectives, and willingness to participate. The sample included physicians, nurses, and ancillary healthcare providers.

Group (2): A convenience sampling technique was employed to select all available oncology patients who fulfilled the following inclusion criteria and agreed to participate in the study: patients receiving care at the Oncology Institution, and being an adult between 18 and 70 years of age. The sample consisted of 60 patients.

### Research instruments

The data for this study were collected using five pretested and validated questionnaires.

I. **Demographic Data Structured Sheet**.

It collects detailed information about the patients’ demographic characteristics. It includes their age, gender, home address, number of years with the disease, use of a smartphone, and mobile apps. Additionally, the sociodemographic data of healthcare workers includes their age, level of education, work shifts, and years worked in their current position.

II. Chatbot Usability Questionnaire.

The Chatbot Usability Questionnaire (CUQ) is a standardized tool designed to evaluate the usability and user satisfaction of chatbot systems. The questionnaire used in this study was adopted from a previously published and validated instrument developed by Holmes and Murgatroyd (2020) [10]. The CUQ has been subsequently used in previous studies, including Nguyen et al. (2024), supporting its applicability and relevance in evaluating chatbot-based health applications [11]. To calculate the CUQ score, first assign each question a score from 1 to 5 based on your agreement (1 = Strongly disagree, 5 = Strongly agree). Sum all odd-numbered (positive) questions and subtract 8, then sum all even-numbered (negative) questions and subtract that total from 40. Add the two results to get a score out of 64, then divide by 64 and multiply by 100 to obtain the CUQ score as a percentage. The internal consistency of the tool was measured using Cronbach’s Alpha, resulting in a score of 0.89, an acceptable level of reliability for research purposes.

III. ** Perceived benefits of healthcare mobile app chatbot tool**.

This tool consists of 8 multiple-choice questions designed to assess the benefits of healthcare chatbot to patients. The tool was developed by Palanica et al. (2019) [12]. Each item reflects a specific benefit, including improved self-management of health, enhanced quality and personalization of care, reduced travel time and unnecessary visits, greater disclosure of information, increased privacy, and better access to timely care. Responses are rated on a 5-point Likert scale ranging from 1 (Strongly Disagree) to 5 (Strongly Agree), with total scores ranging from 8 to 40. Higher scores indicate greater perceived benefits. The questionnaire was translated into Arabic, and both its validity and reliability were assessed. The internal consistency of the tool, measured using Cronbach’s Alpha, was 0.84, indicating an acceptable level of reliability for research purposes.

IV. ** Health awareness efficiency tool of the medical team**.

This tool includes a pre-and post-application evaluation consisting of 10 questions. Each question is rated using a 5-point Likert scale, where (1 = Strongly Disagree, 2 = Disagree, 3 = Neutral, 4 = Agree, and 5 = Strongly Agree). The tool is developed by the researcher based on research conducted by Pan et al. (2022) [9], to measure how increasing patient awareness can influence the medical team’s performance and communication. Higher total scores indicate a stronger perceived impact of health awareness on team efficiency. The total score ranges from 10 to 50, with higher scores indicating a greater perceived impact of health awareness on team efficiency. The scoring system is categorized as follows: a score from 10 to 26 indicates less impact, 27 to 38 reflects a moderate impact, and 39 to 50 signifies a positive impact on the efficiency of the medical team as perceived by the respondents. The questionnaire was translated into Arabic, and both its validity and reliability were assessed. The internal consistency of the tool, measured using Cronbach’s Alpha, was 0.81, indicating an acceptable level of reliability for research purposes.

V. **Effectiveness of the Application in Controlling Chemotherapy Side Effects Tool**.

This tool consists of 8 items with binary response options (1 = No, 2 = Yes). It was developed by the researcher based on reference [7] to assess patients’ perceptions of the effectiveness of the AI-guided application in managing and controlling chemotherapy-related side effects. The tool evaluates whether the application assists patients in monitoring, coping with, and reducing the perceived impact of common chemotherapy side effects. The total score ranges from 8 to 16, with higher scores indicating greater perceived effectiveness of the application in controlling chemotherapy-related side effects.

### Validity and reliability of the instruments

Content validity of all study instruments was assessed by a panel of three experts in Community Health Nursing, who evaluated each item for relevance, clarity, coherence, and simplicity. Necessary modifications were made based on their feedback, confirming that the instruments were suitable for their intended purposes. Additionally, test–retest reliability was evaluated for the study instruments using a subset of participants over a two-week interval. The analysis yielded a stability coefficient of 0.85, representative of good reliability.

### Pilot study

A pilot study was conducted on 10% of the study sample to assess the feasibility of the study, as well as the clarity and objectivity of the tools. The pilot study was excluded from the total study sample size.

### Field work and data collection

Data were collected between August and November 2023. After obtaining approval from the Research and Ethics Committee, all participants were invited to complete a pretest survey via a secure online link using Google Forms. This survey assessed baseline knowledge and competence regarding cancer care protocols prior to the intervention. The study was implemented in three consecutive phases to ensure systematic development, implementation, and evaluation of the AI-based mobile application.

### Phase I: Application design and development

An AI-based mobile application was specifically developed for cancer patients using the Dialogflow platform. The chatbot architecture consisted of predefined intents and entities designed to address common patient needs, including appointment scheduling, treatment preparation, symptom management, medication adherence, nutrition guidance, and self-care practices during chemotherapy. patients were prompted to interact with the chatbot at least once daily, with session durations averaging 5–10 min. The chatbot employed conditional logic, directing responses according to reported symptoms, treatment phase, and user-selected concerns.

Clinical red flags (e.g., severe side effects or emergency symptoms) triggered immediate alerts, advising the user to contact their healthcare provider and providing a summary report to the nurse in charge. Content and reminders were personalized based on patient age, cancer type, treatment stage, and previous interactions. Educational content was structured into thematic modules (e.g., pre-treatment preparation, management of chemotherapy side effects, nutrition during therapy, and follow-up care) and derived from internationally recognized oncology clinical practice guidelines, including those of WHO and the American Cancer Society (ACS). All content was reviewed and validated by oncology nursing and medical experts for accuracy, clarity, and cultural appropriateness.

Prior to full implementation, a two-week pilot test assessed system functionality, navigation flow, clarity of chatbot responses, and overall usability. Feedback from pilot participants resulted in minor modifications, including simplification of medical terminology, refinement of automated reminders, and improved navigation between chatbot options, ensuring standardization of the intervention.

### Phase II: Application implementation

The application was introduced to the target group of cancer patients to support appointment booking, communication with healthcare teams, and access to educational materials. Patients used the chatbot to schedule and confirm appointments, receive automated reminders, and review educational content related to treatment phases and side-effect management. Healthcare workers were simultaneously concerned with to the system to monitor bookings, track patient adherence, and provide additional support when required, simplifying coordinated and continuous care.

### Phase III: Evaluation phase

Subsequent the intervention period, a posttest survey was administered using the same instruments as at baseline. This assessment evaluated improvements in patients’ understanding of care protocols, adherence to scheduled visits, and satisfaction with the application. It also examined the impact of the AI-based mobile application on healthcare workers’ competence in appointment management and patient education. Pretest and posttest findings were analyzed to determine the overall effectiveness of the intervention.

### Statistical analysis

Data were entered and analyzed using the Statistical Package for the Social Sciences (SPSS) version 22. Descriptive statistics, including means, standard deviations, and frequencies, were used to summarize participant characteristics and baseline data. Paired t-tests were conducted to compare pretest and posttest scores for both patients and healthcare workers, while Chi-square tests were used to examine associations between categorical variables, with statistical significance set at *p* < 0.05.

## Results

Table 1a presents the demographic characteristics of the studied patients. The mean age was 37.81 ± 5.89 years. The majority of participants were female (85.0%), and 58.3% had completed secondary education. Most patients (96.7%) had been diagnosed with the disease for more than 5 years. Regarding technology use, 68.3% of patients reported using smartphones to access mobile applications, while the remainder used personal computers or laptops.

**Table 1a:** Distribution of demographic data of studied patients (*n* = 60)

Items		No	%
Age	(Mean ± SD)	37.81 ± 5.89	
Age group	Under 30 years old	18	30.0
	From 30:Under than 40 years old	38	63.3
	over 40 years old	4	6.7
Sex	Male	9	15.0
	Female	51	85.0
Educational level	Basic Education	5	8.3
	Secondary Education	35	58.3
	University Education or above	20	33.3
**Number of years have the disease**	More than 5 years	58	96.7
	Less than 5 years old	2	3.3
**Tools used to have the Mobile Apps**	Personal Computer	15	25.0
	The smartphone	41	68.3
	laptop	4	6.7

Table 1b presents the demographic characteristics of the studied Health Care Workers. The majority of participants were nurses (50.0%) and 48.3% were female. Most participants held a Bachelor’s degree (58.3%), and 41.7% had 6–10 years of professional experience. Regarding work shifts, half of the participants (50.0%) worked rotating shifts (≥ 12 h), while the remainder worked morning/afternoon or night shifts.

**Table 1b:** Distribution of demographic data of studied Health Care Worker (*n* = 60)

Items		No	%
Age group	≤ 20	35	58.3
	21–40	10	16.7
	41–50	15	25.0
**Health Care Worker**	Nurses	30	50.0
	Physicians	20	33.3
	Others health care workers	10	16.7
Sex	Male	31	51.7
	Female	29	48.3
Educational level	Bachelor’s Degree	35	58.3
	Master’s Degree	10	16.7
	Technical Institute	15	25.0
**Years Worked**	Less than 5 years	20	33.3
	From 6 to 10 years	25	41.7
	More than 10 years	15	25.0
**Work Shifts**	Morning/Afternoon	20	33.3
	Nights Only	10	16.7
	Rotating (12 h or more)	30	50.0

Table 2 shows the percentage distribution of Chatbot Usability Questionnaires about the positive effects of the chatbot on providing care for patients from the patient’s point of view. About two-thirds of the studied patients (65.0%, 66.7%, 66.7%) agreed that the chatbot was welcoming during the initial setup, understood them well, and coped well with any errors or mistakes, respectively. 58.3% of the patients agreed that the chatbot responses were useful, appropriate, and informative.

**Table 2:** Percentages distribution of Chatbot Usability Questionnaires about positive effects of the chatbot on providing care of Patient from the patient’s point of view number = 60 patients)

Positive effect	Strongly disagree		Disagree			Neutral			Agree			Strongly agree		
			No	%	No		%	No		%	No		%	
1. The chatbot’s personality was realistic and engaging	0	0.0	13	21.7	25		41.7	22		36.7	0		0.0	
2. The chatbot was welcoming during initial setup	0	0.0	0	0.0	1		1.7	39		65.0	20		33.3	
3. The chatbot explained its scope and purpose well	0	0.0	19	31.7	0		0.0	24		40.0	17		28.3	
4. The chatbot was easy to navigate	0	0.0	26	43.3	2		3.3	22		36.7	10		16.7	
5. The chatbot understood me well	0	0.0	4	6.7	4		6.7	40		66.7	12		20.0	
6. The chatbot responses were useful, appropriate and informative	0	0.0	0	0.0	2		3.3	35		58.3	23		38.3	
7. The chatbot coped well with any errors or mistakes	0	0.0	4	6.7	4		6.7	40		66.7	12		20.0	
8. The chatbot was very easy to us	0	0.0	21	35.0	1		1.7	19		31.7	19		31.7	

Table 3 clarifies the percentage dissemination of Chatbot Usability Questionnaires about the negative effects of the chatbot on providing care for patients from the patient’s point of view (number = 60 patients). More than half of the studied patients (53.3%) agreed that the chatbot would be easy to get confused when using it, that the chatbot responses were irrelevant, and that the chatbot seemed unable to handle any errors. 70.0% of them agreed that the chatbot failed to recognize many of their inputs.

**Table 3:** Percentages distribution of Chatbot Usability Questionnaires about negative effects of the chatbot on providing care of Patient from the patient’s point of view number = 60 patients)

Negative effect	Strongly disagree		Disagree			Neutral			Agree			Strongly agree		
			No	%	No		%	No		%	No		%	
1. The chatbot seemed too robotic	1	1.7	19	31.7	16		26.7	24		40.0	0		0.0	
2. The chatbot seemed very unfriendly	7	11.7	13	21.7	13		21.7	27		45.0	0		0.0	
3. The chatbot gave no indication as to its purpose	8	13.3	29	48.3	0		0.0	23		38.3	0		0.0	
4. It would be easy to get confused when using the chatbot	3	5.0	19	31.7	6		10.0	32		53.3	0		0.0	
5. The chatbot failed to recognize a lot of my inputs	0	0.0	10	16.7	3		5.0	42		70.0	5		8.3	
6. Chatbot responses were irrelevant	6	10.0	13	21.7	9		15.0	32		53.3	0		0.0	
7. The chatbot seemed unable to handle any errors	5	8.3	10	16.7	3		5.0	32		53.3	10		16.7	
8. The chatbot was very complex	5	8.3	15	25.0	3		5.0	27		45.0	10		16.7	

Table 4 presents the distribution of chatbot usability scores among healthcare workers. The total chatbot usability score ranged from 22 to 46, with a mean score of 36.86 ± 6.26. Usability outcomes were additionally expressed as a mean percentage score, which ranged from 34.38% to 71.88%, with an overall mean percentage of 57.60% ± 9.78. This percentage-based presentation provides contextual understanding of the overall usability level perceived by healthcare workers.

**Table 4:** Distribution of Chatbot Usability Scores in Providing Patient Care among Healthcare Workers (*N* = 60)

Items	Min	Max	Mean	SD
Positive effects	22.0	37.0	30.33	4.20
Negative effects	18.0	32.0	25.47	3.69
Total score of Chatbot Usability	22.0	46.0	36.86	6.26
Mean percentage of Chatbot Usability	34.38	71.88	57.60	9.78

Note: The total chatbot usability score is derived from the summed item responses, with possible higher scores reflecting greater perceived usability

Table 5 presents the means and standard deviations of healthcare workers’ perceptions of the benefits of the chatbot for patients (*N* = 60 HCWs). An increase in perceived benefits was observed from pre- to post-intervention. Most items demonstrated a highly statistically significant difference at the 1% level, as assessed using paired t-tests, indicating that HCWs perceived potential improvements in patient care, privacy, accessibility, and personalized treatment. However, these findings reflect perceived changes over time and should be interpreted cautiously due to the quasi-experimental design and the absence of a control group.

**Table 5:** HCWs’ Perceptions of benefits of health care chatbots for patients (*N* = 60 Health Care Workers)

Items	Pre intervention	Post intervention	Paired t-test	*P* value	Effect size (Cohen’s d
1. Help patients better manage their own health	2.78 ± 0.76	3.88 ± 0.94	4.641^HS^	0.000	1.23
2. Improve quality of patient care	3.72 ± 0.94	4.05 ± 0.96	8.600 ^HS^	0.000	0.35
3. Help provide more personalized treatment	3.61 ± 1.09	4.88 ± 1.06	9.442 ^HS^	0.000	1.19
4. Reduce travel time to health care provider	3.63 ± 1.12	4.91 ± 1.07	9.835 ^HS^	0.000	1.13
5. Prevent unnecessary visits to health care providers	2.86 ± 1.02	3.92 ± 1.15	4.252 ^HS^	0.000	0.97
6. Patients may disclose more information to chatbots compared with health care providers	3.71 ± 1.07	4.01 ± 0.99	3.586 ^S^	0.015	0.28
7. Increase patient privacy	2.61 ± 0.95	4.75 ± 0.97	12.01 ^HS^	0.000	2.27
8. Improve access and timeliness to care	3.82 ± 1.016	3.95 ± 0.85	0.756 ^NS^	0.451	0.12

Note: HS = highly statistically significant (*p* < 0.01), S = statistically significant (*p* < 0.05), NS = not statistically significant (*p* ≥ 0.05). Pre- and post-intervention scores were compared using paired t-tests. Effect sizes (Cohen’s d) were calculated for pre–post comparisons to indicate the magnitude of intervention effects (0.2 = small, 0.5 = medium, 0.8 = large)

**Fig. 1:**
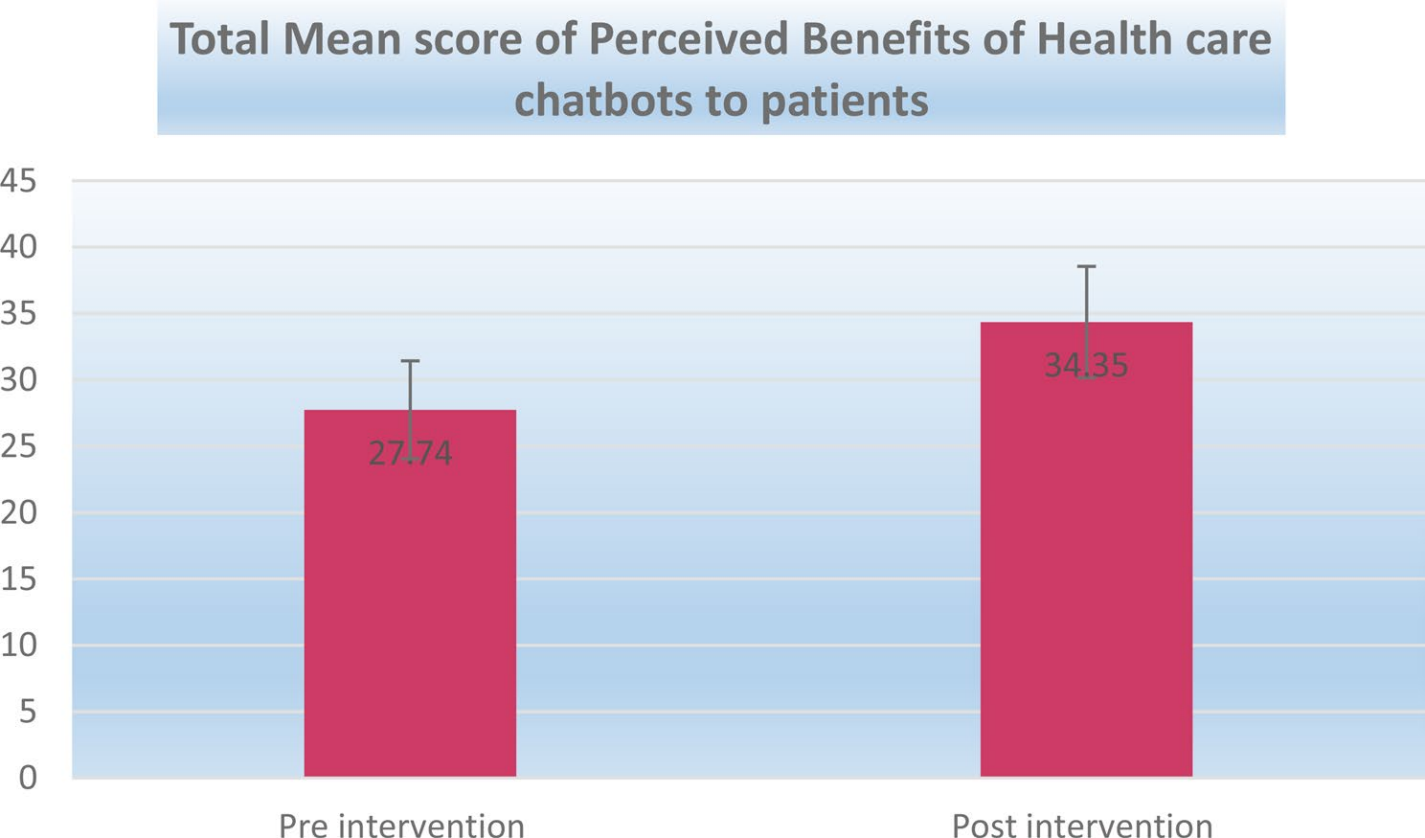
Total mean score of perceived benefits of healthcare chatbot to patients before and after the intervention (60 nurses)

Table 6; Fig. 1 show the total mean score of Perceived Benefits of Health Care Chatbots to patients. It clarifies that the total score of Perceived Benefits of Health Care Chatbots to patients on pre-intervention was 27.74 ± 3.69 and 34.35 ± 4.20 on post-intervention. There was a statistically significant difference between pre and post-intervention at the 1% level of significance. This change reflects an improvement in perceived benefits over time; however, the findings should be interpreted cautiously given the quasi-experimental design and absence of a control group.

**Table 6:** Total mean score of Perceived Benefits of Health care chatbots to patients

Items	Pre intervention	Post intervention	Paired t-test	*P* value	Effect size (Cohen’s)
Total score of Perceived Benefits of Health care chatbots to patients	27.74 ± 3.69	34.35 ± 4.20	8.732^HS^	0.000	1.67

Note: HS: **M**eans highly statistical significance, Effect size (Cohen’s d) calculated for pre–post comparison to indicate magnitude of intervention effect (0.2 = small, 0.5 = medium, 0.8 = large)

Table 7 demonstrates the means and standard deviations assessing the impact of health awareness on the efficiency of medical staff using the chatbot (*N* = 60 nurses). Improved health awareness was observed post-intervention compared to pre-intervention. The differences were highly statistically significant at the 1% level, as determined using paired t-tests.

**Table 7:** The means and standard deviations the of impact of health awareness on the efficiency and medical staff using the chatbot on pre and post application (60 nurses)

Items	Pre intervention	Post intervention	Paired t-test	*P* value
• The health awareness provided by the application contributes to Increasing the efficiency of the service provided to patients, not overburdening them, and bearing the expenses:	2.86 ± 0.85	3.58 ± 1.22	-3.71^HS^	0.000
• Maintaining the safety and safety of the environment in which health care is provided	2.65 ± 0.95	3.31 ± 0.89	-3.95 ^HS^	0.000
• Health awareness through the application contributes to how to use available resources to provide the best service at the lowest cost and improve development economy.	2.18 ± 0.89	3.32 ± 1.36	-5.39 ^HS^	0.000
• Health awareness of hospital management through application contributes to taking into account the expected obstacles and determining how to deal with them or avoided	2.62 ± 0.76	3.45 ± 1.015	-5.08 ^HS^	0.000
• Health awareness through the application contributes to improving the quality of effective nursing interventions and raising the health economy	2.68 ± 0.72	3.53 ± 1.15	-4.82 ^HS^	0.000
• Reducing the consumption of resources used daily within the hospital and thus kills the cost of health consumables spent	2.83 ± 0.56	3.45 ± 0.85	-4.69 ^HS^	0.000
• Improve the application of practical content to patients effectively	2.53 ± 0.67	3.80 ± 1.25	-6.92 ^HS^	0.000
• Health awareness can mobilize and employ various resources Extracting distinguished service by using the human workforce to perform specific roles	2.73 ± 0.89	3.20 ± 1.09	-2.56 ^S^	0.012
• Health awareness through the application contributes to improving current medical problems and how to deal with obstacles that hinder growth economical	2.78 ± 0.69	3.09 ± 0.94	-1.98 ^S^	0.049
• Reduce the use of paper effort	2.60 ± 0.81	3.71 ± 0.95	-1.93 ^S^	0.043
Total	26.48 ± 3.41	33.48 ± 5.89	-7.96 ^HS^	0.000

Note: HS = highly statistically significant (*p* < 0.01), S = statistically significant (*p* < 0.05), NS = not statistically significant (*p* ≥ 0.05). Pre- and post-intervention scores were compared using paired t-tests

**Fig. 2:**
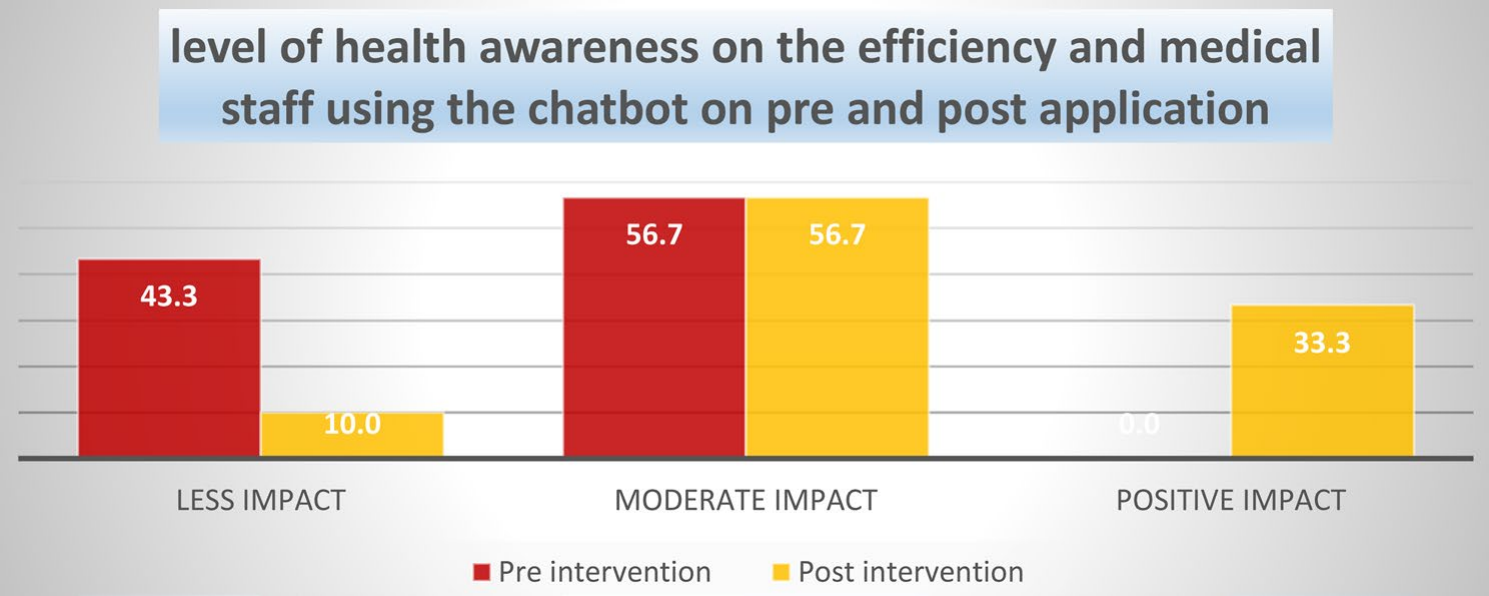
Levels of health awareness related to efficiency among medical staff using the chatbot before and after application (*N* = 60 nurses). Note: “Less impact,” “moderate impact,” and “positive impact” represent increasing levels of perceived effectiveness based on the total scale score

Figure 2 illustrates participants’ health awareness regarding the efficiency and use of the chatbot by medical staff, pre- and post-intervention. At baseline, 0% of participants were classified in the positive impact category, reflecting a low initial level of health awareness. Following the intervention, 33.3% of participants reached the positive impact category, indicating an improvement. Classification thresholds for the summed scale were applied consistently across both time points.

**Fig. 3:**
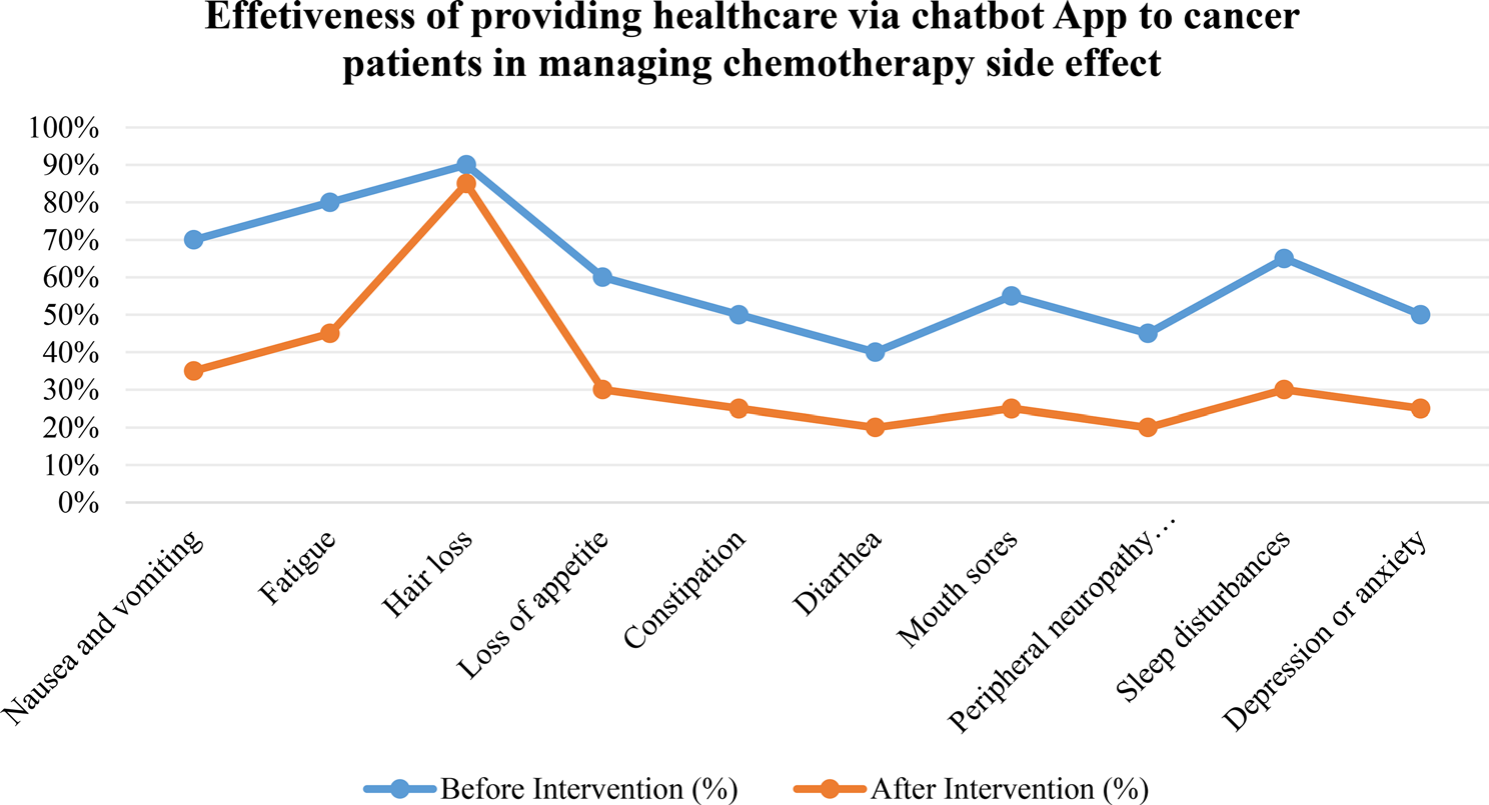
Effectiveness of the AI-powered application in controlling chemotherapy-related side effects among patients before and after intervention

Figure 3 presents the distribution of chemotherapy-related side effects among the studied patients before and after intervention. The figure shows a descriptive reduction in the reported prevalence of several side effects following the intervention. Prior to application use, higher levels of symptoms were reported, including nausea and vomiting (70%), fatigue (80%), loss of appetite (60%), constipation (50%), diarrhea (40%), mouth sores (55%), peripheral neuropathy (45%), sleep disturbances (65%), and depression or anxiety (50%). Following the intervention, the reported prevalence of these symptoms decreased to 35% for nausea and vomiting, 45% for fatigue, 30% for loss of appetite, 25% for constipation, 20% for diarrhea, 28% for mouth sores, 22% for peripheral neuropathy, 30% for sleep disturbances, and 28% for depression or anxiety. These findings describe observed trends in symptom reporting before and after application use. Although substantial reductions in chemotherapy-related side effects were observed post-intervention, the lack of a control group means that these improvements may be influenced by time, concurrent care, rather than solely by the AI-guided application.

Table 8 displays a significant improvement in nurses’ health awareness regarding the efficiency of using the chatbot after the intervention. Before the application, 43.3% of nurses reported less impact and none stated a positive impact, whereas after the intervention, the percentage of less impact reduced to 10.0% and 33.3% reported a positive impact. The Chi-square test indicated a significant difference between pre- and post-intervention results (χ² = 32.50, *p* < 0.001), confirming the effectiveness of intervention in improving medical staff awareness and perceptions.

**Table 8:** The level of health awareness on the efficiency and medical staff using the chatbot on pre and post application (60 nurses)

Level	Pre intervention		Post intervention	
	No	%	No	%
Less impact	26	43.3	6	10.0
Moderate impact	34	56.7	34	56.7
Positive impact	0	0.0	20	33.3
X^2^	32.50^HS^			
p-value	0.000			

## Discussion

AI has revolutionized healthcare service delivery, especially in chronic disease management, through its integration into mobile health applications. AI-based mobile application offer personalized preventive and treatment approaches, empowering patients in self-management, therapy adherence, and timely advice receipt. In cancer, digital interventions have the potential to enhance treatment outcomes, self-management, and alleviate healthcare system burdens. AI-based tools may enhance healthcare workers’ competence by providing decision support, real-time patient data, and evidence-based recommendations. This study explores the effectiveness of AI-based mobile application in improving patient outcomes and their impact on the skills and confidence of healthcare providers.

In this study, the majority were female, reflecting gendered health-seeking behaviors and AI-based mobile application usage trends. Regarding education, 58.3% had secondary education and 33.3% had university-level or higher education, indicating sufficient literacy to engage effectively with an AI-based mobile application. The numerousness of younger, educated females may have contributed to the positive insights of usability and engagement observed. However, these demographic characteristics decrease the generalizability of the findings to older, less educated, or male populations. Future studies should aim to include more diverse patient samples to assess the applicability and efficacy of AI-guided interventions across larger demographic group.

This results consistent with studies by Melhem, (2023) [13] and Moon, & Walsh. (2025) [14], which report higher engagement among women and literate populations. The high proportion of patients living with chronic illness for over five years further supports receptiveness to AI-guided interventions, echoing findings that longer disease duration correlates with greater digital health engagement Ezeigwe et al., 2025 [15].

The demographic characteristics of the healthcare workers in this study align closely with findings reported by Khan Rony et al. (2024) [16], who reported that mid-career healthcare professionals demonstrate the highest readiness and adaptability to digital innovations compared with younger or pre-retirement staff. The high percentage of workers holding a Bachelor’s degree corresponds with findings from Ventura et al. (2023) [17], who noted that higher educational attainment significantly enhances digital competence and facilitates acceptance of AI-based applications.

The results indicated that the chatbot was generally perceived as usable and well-received by patients, reflecting participants’ perceptions of potential support in managing their care. Patients reported that the chatbot facilitated engagement and adherence to treatment protocols, although some usability challenges were noted. These findings are partially consistent with prior studies demonstrating that AI-based mobile application can enhance patient engagement, adherence, and access to personalized health information Chang et al., 2025 [18]. However, differences in stated usability issues, such as input recognition errors, highlight the influence of interface design, participant digital literacy, and context-specific factors. Similarly, healthcare workers reported perceived enhancements in clinical communication and patient support, corroborating previous findings that structured exposure and training may increase confidence and competence in digital health technologies Wang et al., 2023 [19]. It should be noted that, as this study lacked a control group and relied on self-reported measures, observed improvements should be interpreted as suggestive rather than definitive evidence of effectiveness.

Notably, this study found that AI-based mobile application usage was associated with observed improvements in the management of chemotherapy-related side effects, including fatigue, nausea, hair loss, and sleep disturbances. The AI-based mobile application assisted patients in managing chemotherapy-related side effects, including alopecia, by providing education, coping strategies, and emotional support. Patients reported feeling more prepared and psychologically supported in dealing with hair loss and other treatment-related symptoms. Similarly, a recent pilot study using a text messaging integrated, chatbot‑interfaced system for gastrointestinal cancer patients undergoing chemotherapy reported that patients found the system user‑friendly and valuable for self‑management; the intervention was associated with increased patient activation and better symptom control Gomaa et al., 2023 [20]. However, as this was a single-arm study without a control group, observed improvements may also be influenced by time, expectation, or concurrent clinical care.

Overall, the findings strengthen the value of AI-based mobile application and chatbot in observed improvements in patient outcomes and healthcare worker performance. They further suggest that integrating AI-based interventions into both patient care and professional training programs can strengthen digital health literacy, promote patient-centered care, and reduce healthcare system burdens, consistent with the broader literature on AI in healthcare Aggarwal et al., 2023 [21].

The results of the current study reveal that the use of an AI-based mobile application was associated with an increase in patient care by observed improvements in adherence to prevention and treatment protocols, potentially reducing chemotherapy-related side effects, and increasing healthcare workers’ efficiency. Similarly, the study conducted by Greer et al. (2019) [22] demonstrated that chatbots provide emotional support, improve patient engagement, and facilitate symptom monitoring among cancer survivors. Moreover, recent research by Gomaa et al. (2023) [20], reported that AI-based mobile application enhanced self-management, symptom control, and patient activation in oncology settings. However, given the single-arm design and reliance on self-reported measures in the current study, these observed associations should be interpreted as suggestive rather than conclusive evidence of causality [23–26]. Recent evidence highlights that the COVID-19 pandemic has accelerated the adoption of telemedicine and digital health technologies across various patient populations, including cancer and cardiovascular disease patients [27, 28]. These studies suggest that widespread exposure to remote care has improved digital health literacy, increased patient trust in virtual interventions, and normalized engagement with technology-mediated healthcare. AI-guided mobile application was associated with improvements in healthcare workers’ perceptions, patient health awareness, and self-reported management of chemotherapy-related side effects. While these findings are promising, they should be interpreted with caution. The quasi-experimental design without a control group, the reliance on self-reported measures, and the potential influence of external factors such as time and concurrent care limit the ability to make definitive causal conclusions.

The results showed that the most frequent interventions were related to telemedicine and data management, while gaps existed in individual-based data reporting during the pandemic. This highlights the need for patients to take a more active role in managing their health. Although positive outcomes were observed with the AI-based mobile application, sustained patient engagement such as contributing PROMs or participating in digital registries depends on motivation, digital literacy, and trust in data governance. Differences in technological competence and potential digital fatigue may limit long-term adoption. Future research should explore patients’ readiness for active involvement to support effective implementation of digital health interventions.

### Limitations of the study

This study has several limitations. First, purposive sampling of healthcare workers and convenience sampling of patients may limit the generalizability of the results. Second, data collection relied on self-reported measures, which may introduce response bias and may oversimplify complex experiences, particularly for tools using binary responses for chemotherapy side effects. Some instruments, including the health awareness efficiency and side effect management tools, were adapted or researcher-developed; however, content validity was confirmed by expert review and test-retest reliability was assessed where applicable. Potential measurement biases remain. Third, the quasi-experimental pre–post design without a control group limits causal inference. Observed changes may be influenced by external factors, such as time or institutional changes, rather than the intervention alone. Fourth, although demographic characteristics of participants were described, their potential influence on intervention outcomes was not analyzed. Future studies should consider stratifying results by age, education, or professional experience to explore possible moderating effects and enhance the generalizability of findings. Finally, this study evaluated short-term outcomes over a 12-week period. The long-term effects of AI-based mobile intervention on patient outcomes and healthcare workers’ competence were not assessed. Future studies should address these limitations by integrating objective outcome measures, such as clinic attendance records, medication adherence data, and supervisor-rated competence assessments. Additionally, research employing larger samples, appropriate control groups, and longer follow-up periods to confirm the present findings.

### Recommendations

Healthcare organizations should prioritize the implementation of AI-based mobile applications as part of routine cancer care to enhance service accessibility, patient safety, and adherence to treatment protocols. In addition, continuous training programs should be provided for healthcare workers to ensure effective use of AI tools and maximize their potential in patient care. Patient engagement strategies should also be developed to increase comfort and proficiency in using AI applications for self-management and remote care. Furthermore, future research should explore integrating AI applications with other healthcare technologies to further optimize patient outcomes and workflow efficiency.

## Conclusion

The integration of artificial intelligence (AI) mobile applications into cancer care was associated with positive effects on both patients and healthcare providers. AI-guided prevention and treatment protocols improve patients’ access to healthcare services, enhance adherence to medical instructions, and contribute to safer, more efficient care. These findings suggest that AI-based mobile application may be promising tools for supporting patient care and enhancing healthcare staff performance. For healthcare providers, AI tools may help reduce patient overcrowding, streamline workflow, and support professional competence. Overall, AI applications appear to be valuable in optimizing cancer care delivery and potentially improving patient outcomes, though further controlled studies are needed to confirm these preliminary observations.

## Data Availability

The dataset used for this study is not publicly available due to the possibility of compromising individual privacy but is available from the corresponding author on reasonable request.
